# The Possible Role of Mast Cells in the Odontogenic Cyst's Pathogenesis: A Comparative Study between Dentigerous Cyst and Keratocystic Odontogenic Tumor

**DOI:** 10.1155/2016/8754567

**Published:** 2016-02-24

**Authors:** Sareh Farhadi, Fatemeh Shahsavari, MirMahdi Davardan

**Affiliations:** ^1^Oral & Maxillofacial Pathology Department, Islamic Azad University, Dental Branch of Tehran, Tehran 16686 49911, Iran; ^2^Islamic Azad University, Dental Branch of Tehran, Tehran 16686 49911, Iran

## Abstract

*Background*. Recently, mast cells were recognized in the pathogenesis of more aggressive pathologic lesions. This study was aimed to evaluate and compare the density of mast cells in Dentigerous cyst (DC) and Keratocystic odontogenic tumor (KCOT) regarding their different clinical behavior.* Method*. This study was conducted on 23 and 26 cases of DC and KCOT, respectively. Four-micron sections were prepared for Toluidine blue staining and mast cell densities in two desired cysts were studied. Final data was analyzed via *t*-test and Mann-Whitney *U* test method regarding the significant level lower than 0.05.* Results*. Mast cell densities were significantly higher in KCOTs for deep and superficial layers and both layers (*P* < 0.05). The density of degranulated mast cells in the deeper layers and both layers was significantly higher in KCOTs (*P* < 0.05). However, the density of degranulated mast cells in the superficial layer had no significant difference (*P* > 0.05).* Conclusion*. It seems that mast cells may be involved in the pathogenesis of KCOT, but, regarding wide range of mast cell's biologic activities, further investigations are recommended to confirm the issue and prepare the details.

## 1. Introduction

Dentigerous cyst is the most common developmental odontogenic cyst with excellent prognosis and low recurrence [1]. Keratocystic odontogenic tumor was described first time by Philipsen in 1956 [2] as an odontogenic cyst. Recently, due to aggressive behavior and tendency to high recurrence of these lesions, the new classification of WHO placed it in the category of the benign odontogenic tumor [3–5] which can invade into cranial, zygomatic, and orbital bone or conflict sinus [6].

Several types of cells are associated with the development of cysts and tumors. Many efforts have been made to understand the pathogenesis of odontogenic cysts, but many of them have been unsuccessful [7]. Several mechanisms have been proposed for the growth and expansion of odontogenic cysts; however, the exact mechanism of enlargement is not known [8]. Recently, among inflammatory cells, mast cells have been considered. First, these cells were described by Paul Ehrlich in 1877 with Mastzellan [9]. These cells are elliptical to circular with a diameter of about 20 to 30 micrometers and are containing basophilic secretory granules of 0.5 to 0.2 micrometers. These secretory cells diffuse around blood vessels, nerves, and lymph throughout the body, but especially in the skin; digestive mucosa and respiratory systems can be seen in abundance [10–12]. Mast cells can have an inhibitory role on the development of pathological lesions. However, stimulatory role of mast cells in the growth of pathological lesions is more prevalent and obvious than their inhibitory effect [13]. It seems that the stimulatory role depends on many factors and conditions.

The mast cell granules contain numerous active components as histamine, tryptase, and kinase with stimulatory or inhibitory effects. Also, some of them, such as heparin, would be able to present both stimulatory and inhibitory agents for microenvironment signalling pathway [14]. On the other hand, the other mediators produced by stromal cells adjacent to mast cells as fibroblasts and endothelial cells may potentially influence the pathogenesis of the lesions through a variety of mechanisms such as cyclooxygenase metabolites and heparinizes [14].

Mast cells may be associated with the pathogenesis of cysts too. Previous studies have identified mast cells in odontogenic cysts [8], but there were limited studies about the role of mast cells in the pathogenesis of odontogenic cysts. So, this study was aimed to evaluate and compare the density of mast cells in Dentigerous cysts (DC) and Keratocystic odontogenic tumor (KCOT).

## 2. Material and Methods

This experimental study evaluates the pathologic records of patients with final diagnosis of Dentigerous cyst and Keratocystic odontogenic tumor from the year 2006 to the year 2014 selected from the archive of Oral Pathology Department, Dental Branch of Tehran, Islamic Azad University, Tehran, Iran. 49 cases include 23 cases of Dentigerous cyst, and 26 cases of Keratocystic odontogenic tumor were chosen after the evaluation of paraffin blocks and related slides. The samples with no sufficient tissue for microscopic evaluation, bleeding, and/or necrosis were excluded from the study.

**Figure 1:**
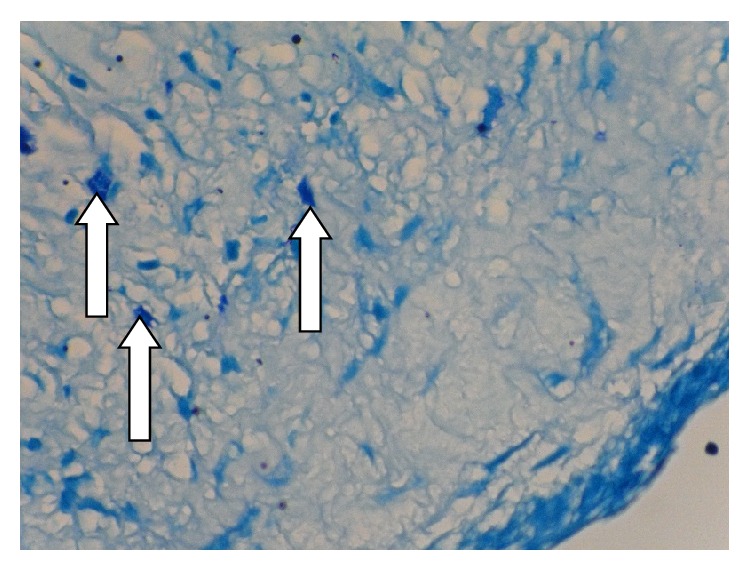
Mast cells (arrow tip) in a deep area of Dentigerous cyst wall stained with Toluidine blue by 400x magnification.

**Figure 2 fig2:**
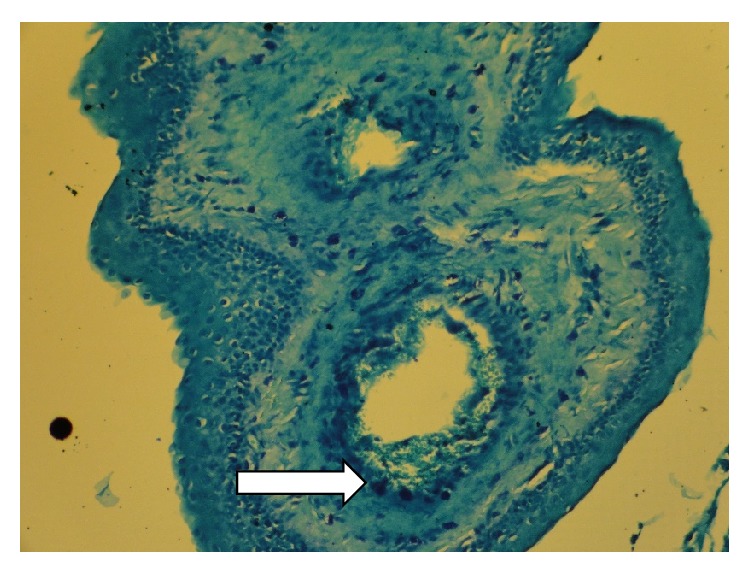
Many mast cells in the wall of Keratocystic odontogenic tumor stained with Toluidine blue by 200x magnification; one of them marks with arrow tip.

**Figure 3 fig3:**
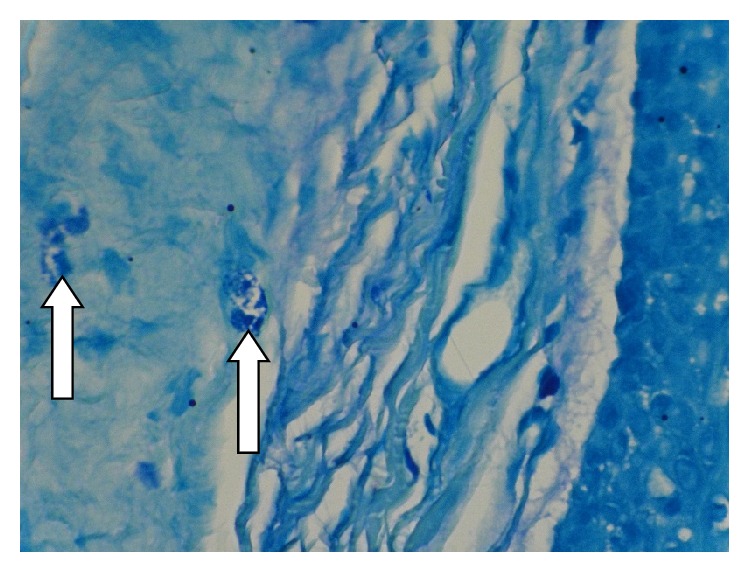
Degranulated mast cells (arrow tip) in the superficial area of the Keratocystic odontogenic tumor wall, stained with Toluidine blue by 400x magnification.

**Figure 4:**
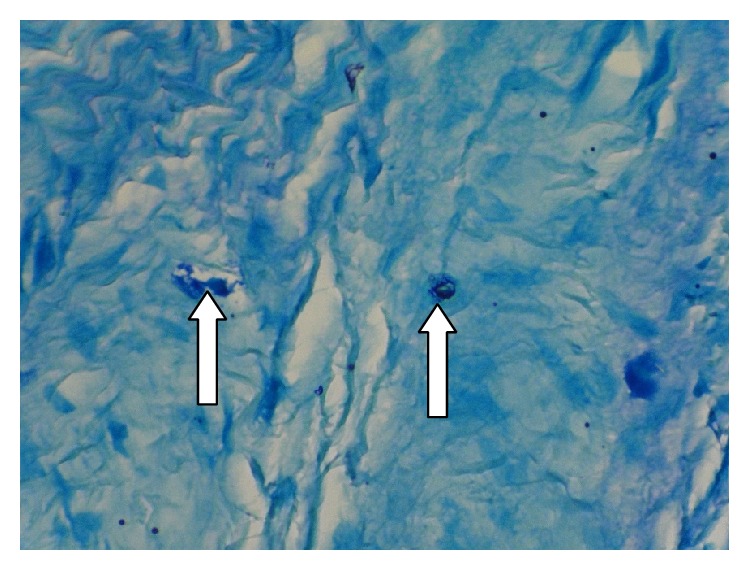
Degranulated mast cells (arrow tip) in a deep area of Dentigerous cyst wall stained with Toluidine blue by 400x magnification.

Four-micron sections from the selected blocks were prepared for Toluidine blue staining to assess and count mast cell. Each slide was examined by two observers by Olympus optical microscope with a magnification of 400 times. For each sample, the granulated mast cells were counted in five view of the deep layer and five view of the superficial layer and also the degranulated mast cells were counted again in five view of the deep layer and five view of the superficial layer. Then, the number of granulated and degranulated mast cells in the total of two layers and also the total number of mast cells in both layers were calculated and the average amounts of the counted cells were recorded (Figures 1 [Fig fig2] [Fig fig3]–4). The extracting data was analyzed using statistical software SPSS Version 22 via *t*-test otherwise Mann-Whitney *U* test and significant level of 0.05 was set.

## 3. Results

As Table 1 showed, the densities of mast cells in the deep layer of Dentigerous cyst and Keratocystic odontogenic tumor were 8 ± 5 and 23 ± 10, respectively. On the other hand, the densities of degranulated mast cells in the deep layer of the Dentigerous cyst and Keratocystic odontogenic tumor were 1 ± 2 and 2 ± 2, respectively.

Also, the densities of mast cells in the superficial layer of Dentigerous cyst and Keratocystic odontogenic tumor were 8 ± 5 and 16 ± 8, respectively. Furthermore, the densities of degranulated mast cells in the superficial layer of Dentigerous cyst and Keratocystic odontogenic tumor were 0 ± 1 and 1 ± 1, respectively. Finally, the densities of degranulated mast cells in both layers of Dentigerous cyst and Keratocystic odontogenic tumors were 2 ± 2 and 2 ± 3, respectively.

According to Table 2, the density of mast cells in deep and superficial layer and both layers of Dentigerous cyst and Keratocystic odontogenic tumor had significant differences, presenting that the density of mast cells was higher in all cases of Keratocystic odontogenic tumor (*P* value < 0.05).

Regarding Table 3, the densities of degranulated mast cells in deep layer and both layers in Dentigerous cyst and Keratocystic odontogenic tumor have significant differences, presenting that the density of mast cells was higher in all cases of Keratocystic odontogenic tumor (*P* value < 0.05). But the density of degranulated mast cells in the superficial layer of Dentigerous cyst and Keratocystic odontogenic tumor had not significant differences (*P* value > 0.05).

## 4. Discussion

The present study showed that the average density of mast cells in deep and superficial layer and both layers was significantly higher in odontogenic keratocyst versus Dentigerous cyst (*P* value < 0.05). Also, the average density of degranulated mast cells in the deep layer and both layers was significantly higher in Keratocystic odontogenic tumors versus Dentigerous cyst (*P* value < 0.05). But the average density of degranulated mast cells in superficial layer of Dentigerous cyst and Keratocystic odontogenic tumors had no significant difference (*P* value > 0.05).

In the study of de Noronha Santos Netto et al., in 2012, inflammatory Dentigerous cysts and Keratocystic odontogenic tumors have more average of mast cells as compared to noninflammatory lesions in all sectors. Along with this present study, deep sections of all cysts showed greater average of degranulated mast cells [8]. On the other hand, in the study of Teronen et al., in 1996, compared with the results of the present study, the majority of mast cells had been detected in inflammatory areas and just below the epithelium of cyst, but the majority of mast cells in periphery of the lesion have been degranulated [15]. Also, Rajabi-Moghaddam et al. could not find any significant difference between DC, RC, and KCOTs. Their little sample volume might be the notable explanation for this result [16].

Smith et al., in 1989, showed that the density of mast cells had significant difference in keratinized and nonkeratinized odontogenic cysts. They concluded that mast cells participated in inflammatory procedures in nonkeratinized cysts, with inverse action in keratinized cysts [17]. Patidar et al., in 2012, reported the highest number of mast cells in radicular cyst and the lowest number for KCOT, in contrast to the present results presenting subepithelial areas of all cysts with more mast cells compared to deeper areas [7].

Prior to this, the presence of mast cells in odontogenic cysts and tumors, especially in the periapical and Dentigerous cysts and Keratocystic odontogenic tumors, was reported [17]. Also, it was suggested previously that degranulation productions of mast cells can correlate with the increase of extracellular matrix destruction in cystic wall by stimulating cytokine production and thus can facilitate the expansion of the lesion [18]. Regarding the results of the studies of Teronen et al. [15] and de Noronha Santos Netto et al. [8], the presence of mast cells specifically in the wall of connective tissue and also in close proximity with the inflammatory cells resulted in the participation of these cells in the expansion of cyst by the secretion of heparin and other hydrolytic enzymes. This procedure was accompanied by dissolution facilitates of glycosaminoglycan and proteoglycan in the cystic fluid conduit with the increase of osmotic and hydrostatic pressure [19, 20]. Also degranulated mast cells release tryptases and prostaglandins, two substances that can participate in bone loss in the joint line between the cyst and bone, which results in the growing cyst [18].

Regarding the present results, higher density of mast cells was observed in the deepest area of the cyst wall in both lesions. This result may show more activities of these cells in the outer layer of the cystic wall with close vicinity of perilesional bone. It is suggested that these cells may relate to bone loss phenomenon similar to osteoporosis and mastocytosis [21]. Mast cells have been known to participate in bone resorption in the pathogenesis of osteoporosis. Also, it has been shown that these cells can adjust osteoclast activity through releasing their granule related to stem cells factor [20]. Furthermore, it has been shown that these cells can increase bone loss with the production of heparin and TNF-A and can stimulate osteoclast activity [22].

This information accompanied by evidences from the results of the present study suggests that mast cells can affect the pathogenesis of Odontogenic cysts, contributing to bone destruction and cystic growth [15]. So, it seems that the presence and degranulation of mast cells may explain the growth potential of Keratocystic odontogenic tumors. However, the accomplishment of further studies is recommended to illustrate the details.

## 5. Conclusion

The results of this study suggest that mast cells may be involved in the pathogenesis of Keratocystic odontogenic tumors, but, regarding wide range of mast cell's biologic activities, further investigations are recommended to confirm the issue and prepare the details.

## Figures and Tables

**Table 1:** Mast cell densities in all layers of cyst wall of Keratocystic odontogenic tumors and Dentigerous cysts.

Mast cell density (mean ± SD)	Cyst type	
	Dentigerous cyst	Keratocystic odontogenic tumor
Density of mast cells in deep layer	8 ± 5	23 ± 10
Density of degranulated mast cells in deep layer	1 ± 2	2 ± 2
Density of mast cells in superficial layer	8 ± 5	16 ± 8
Density of degranulated mast cells in superficial layer	0 ± 1	1 ± 1
Density of degranulated mast cells in both of the two layers	2 ± 3	2 ± 2

**Table 2:** Comparative results of mast cells densities in Dentigerous cyst and Keratocystic odontogenic tumor.

Statistical points	Mast cell densities					
	Deep layer		Superficial layer		Both layers	
*P* value	0.000	0.000	0.000	0.000	0.000	0.000
Mean difference	14.30		8.32		22.47	

**Table 3:** Comparative results of Mann Whitney *U* test for degranulated mast cells in the superficial and deep layers and both layers.

Statistical points	Degranulated mast cell densities		
	Deep layer	Superficial layer	Both layers
*P* value	0.025	0.628	0.031

## References

[1] Deboni M. C. Z., Brozoski M. A., Traina A. A., Acay R. R., da Graça Naclério-Homem M. (2012). Surgical management of dentigerous cyst and keratocystic odontogenic tumor in children: a conservative approach and 7-year follow-up. *Journal of Applied Oral Science*.

[2] Philipsen H. P. (1956). On keratocysts in the jaws. *Tandleagebladet*.

[3] Neville B., Damm D., Allen C., Bouquot J. (2009). *Oral & Maxillofacial Pathology*.

[4] Forssell K. (1980). The primordial cyst. A clinical and radiographic study. *Proceedings of the Finnish Dental Society*.

[5] Madras J., Lapointe H. (2008). Keratocystic odontogenic tumour: reclassification of the odontogenic keratocyst from cyst to tumour. *The Journal of the Canadian Dental Association*.

[6] Williams T. P., Connor F. A. (1994). Surgical management of the odontogenic keratocyst: aggressive approach. *Journal of Oral and Maxillofacial Surgery*.

[7] Patidar K. A., Parwani R. N., Wanjari S. P., Patidar A. P. (2012). Mast cells in human odontogenic cysts. *Biotechnic and Histochemistry*.

[8] de Noronha Santos Netto J., Pires F. R., da Fonseca E. C., Silva L. E., de Queiroz Chaves Lourenço S. (2012). Evaluation of mast cells in periapical cysts, dentigerous cysts, and keratocystic odontogenic tumors. *Journal of Oral Pathology and Medicine*.

[9] Kessler D. A., Langer R. S., Pless N. A., Folkman J. (1976). Mast cells and tumor angiogenesis. *International Journal of Cancer*.

[10] Glowacki J., Mulliken J. B. (1982). Mast cells in hemangiomas and vascular malformations. *Pediatrics*.

[11] Poole T. J., Zetter B. R. (1983). Stimulation of rat peritoneal mast cell migration by tumor-derived peptides. *Cancer Research*.

[12] Roche W. R. (1986). The nature and significance of tumour-associated mast cells. *Journal of Pathology*.

[13] Coussens L. M., Raymond W. W., Bergers G. (1999). Inflammatory mast cells up-regulate angiogenesis during squamous epithelial carcinogenesis. *Genes and Development*.

[14] Samoszuk M., Kanakubo E., Chan J. K. (2005). Degranulating mast cells in fibrotic regions of human tumors and evidence that mast cell heparin interferes with the growth of tumor cells through a mechanism involving fibroblasts. *BMC Cancer*.

[15] Teronen O., Hietanen J., Lindqvist C. (1996). Mast cell-derived tryptase in odontogenic cysts. *Journal of Oral Pathology & Medicine*.

[16] Rajabi-Moghaddam M., Abbaszadeh-Bidokhty H., Bijani A. (2015). Comparison of mast cells count in odontogenic cysts using histochemical staining. *Iranian Journal of Pathology*.

[17] Smith G., Smith A. J., Basu M. K. (1989). Mast cells in human odontogenic cysts. *Journal of Oral Pathology and Medicine*.

[18] Chatterjee S., Mahajan S., Boaz K., George T. (2008). Quantitative role of mast cells in odontogenic cystic enlargement. *Brazilian Journal of Oral Sciences*.

[19] Smith G., Smith A. J., Browne R. M. (1984). Glycosaminoglycans in fluid aspirates from odontogenic cysts. *Journal of Oral Pathology*.

[20] Smith G., Smith A. J., Browne R. M. (1988). Histochemical studies on glycosaminoglycans of odontogenic cysts. *Journal of Oral Pathology*.

[21] Chiappetta N., Gruber B. (2006). The role of mast cells inosteoporosis. *Seminars in Arthritis and Rheumatism*.

[22] Dražić R., Sopta J., Minić A. J. (2010). Mast cells in periapical lesions: potential role in their pathogenesis. *Journal of Oral Pathology and Medicine*.

